# Identification and profiling of novel microRNAs in the *Brassica rapa* genome based on small RNA deep sequencing

**DOI:** 10.1186/1471-2229-12-218

**Published:** 2012-11-19

**Authors:** Bumjin Kim, Hee-Ju Yu, Sin-Gi Park, Ja Young Shin, Mijin Oh, Namshin Kim, Jeong-Hwan Mun

**Affiliations:** 1 Department of Agricultural Biotechnology, National Academy of Agricultural Science, Rural Development Administration, 150 Suin-ro Gwonseon-gu, Suwon, 441-707, Korea; 2 Department of Life Sciences, The Catholic University of Korea, 43 Jibong-ro Wonmi-gu, Bucheon, 420-743, Korea; 3 Korean Bioinformation Center, Korea Research Institute of Bioscience and Biotechnology, 125 Gwahak-ro Yuseong-gu, Daejeon, 305-806, Korea

**Keywords:** *Brassica rapa*, Genome, miRNA, miRNA target, Small RNA sequencing, Database

## Abstract

**Background:**

MicroRNAs (miRNAs) are one of the functional non-coding small RNAs involved in the epigenetic control of the plant genome. Although plants contain both evolutionary conserved miRNAs and species-specific miRNAs within their genomes, computational methods often only identify evolutionary conserved miRNAs. The recent sequencing of the *Brassica rapa* genome enables us to identify miRNAs and their putative target genes. In this study, we sought to provide a more comprehensive prediction of *B. rapa* miRNAs based on high throughput small RNA deep sequencing.

**Results:**

We sequenced small RNAs from five types of tissue: seedlings, roots, petioles, leaves, and flowers. By analyzing 2.75 million unique reads that mapped to the *B. rapa* genome, we identified 216 novel and 196 conserved miRNAs that were predicted to target approximately 20% of the genome’s protein coding genes. Quantitative analysis of miRNAs from the five types of tissue revealed that novel miRNAs were expressed in diverse tissues but their expression levels were lower than those of the conserved miRNAs. Comparative analysis of the miRNAs between the *B. rapa* and *Arabidopsis thaliana* genomes demonstrated that redundant copies of conserved miRNAs in the *B. rapa* genome may have been deleted after whole genome triplication. Novel miRNA members seemed to have spontaneously arisen from the *B. rapa* and *A. thaliana* genomes, suggesting the species-specific expansion of miRNAs. We have made this data publicly available in a miRNA database of *B. rapa* called BraMRs. The database allows the user to retrieve miRNA sequences, their expression profiles, and a description of their target genes from the five tissue types investigated here.

**Conclusions:**

This is the first report to identify novel miRNAs from *Brassica* crops using genome-wide high throughput techniques. The combination of computational methods and small RNA deep sequencing provides robust predictions of miRNAs in the genome. The finding of numerous novel miRNAs, many with few target genes and low expression levels, suggests the rapid evolution of miRNA genes. The development of a miRNA database, BraMRs, enables us to integrate miRNA identification, target prediction, and functional annotation of target genes. BraMRs will represent a valuable public resource with which to study the epigenetic control of *B. rapa* and other closely related *Brassica* species. The database is available at the following link: http://bramrs.rna.kr [1].

## Background

Most flowering plants have undergone genome duplications during their evolution. Sequencing plant genomes has revealed that most eudicot genomes descended from an ancient hexaploid ancestor and then underwent lineage-specific whole genome duplication (polyploidization). Although this polyploidization has not occurred in grape [2], it has occurred once in poplar [3], legumes [4,5], and potato [6], and twice in *Arabidopsis*[7] and *Brassica*, the latter of which has undergone additional whole genome triplication (WGT) since its divergence from the *Arabidopsis* lineage [8,9]. Polyploidy results in the accumulation of homologous genes in the genome leading to increased complexity and redundancy. Redundant copies of amplified genes in the duplicated genome can diverge soon after duplication, and they might become pseudogenes (nonfunction), or gain additional or novel functions (subfunction and neofunction, respectively). In addition, duplicated genes may play a role in preventing potential harmful mutations (buffering). Regardless of their alterations, the ultimate fate of duplicated genes vary according to the individual plant and species [10,11]. Recent studies of several species have demonstrated that a broad range of genetic and epigenetic responses also occurred soon after polyploidization, including DNA deletions, chromosome rearrangements, cytosine methylation, gene silencing, the activation of transposons, and the modification of parental imprinting [12-15]. These events have been associated with small RNAs [16], indicating that changes in the small RNAs of polyploidy genomes provide insight into the control of the genetic and epigenetic mechanisms that occur in response to genome duplication.

Small RNAs are short noncoding RNAs typically 19–25 nucleotides (nt) in length with two prominent sizes of 21 and 24 nt. In general, microRNAs (miRNAs) correspond to the 21 nt class of small RNAs and short-interfering RNAs (siRNAs) comprise the 24 nt class. Small RNAs have been shown to be involved in a broad range of functions including heterochromatin formation, DNA methylation, and gene silencing [17,18]. By targeting genes for cleavage or repressing protein translation, plant miRNAs play an essential role in various biological and metabolic processes, including tissue identification, developmental control, and response to environmental stress. In contrast, siRNAs seem to function as guardians against transposable elements during plant development [12,19-22]. While both classes of small RNAs have been characterized, recent studies have focused mainly on miRNAs because they regulate diverse developmental processes through the direct cleavage of target messenger RNA (mRNA). The biogenesis of miRNA takes place in a stepwise manner. miRNA is initially transcribed by RNA polymerase II. A long primary transcript (pri-miRNA), which forms a hairpin-like secondary structure, is then processed by the nuclear localized Dicer-like 1 (DCL1) in the plant nucleus to release a 60~70 nt intermediate. The intermediate, known as precursor miRNA (pre-miRNA), has a hairpin structure with base-pairing that is not perfectly complementary, resulting in many mismatches and bulges that are further processed into a miRNA/miRNA* duplex. After undergoing 3’-methylation, a paired set of miRNAs are then exported from the nucleus to the cytoplasm by HASTY [19]. Subsequently, the mature miRNA is loaded onto the RNA-induced silencing complex (RISC) and guides the RISC to recognize complementary sites on target mRNAs, resulting in transcript cleavage [22-24] or translational repression [25,26].

Many miRNAs have been identified by computational or experimental approaches in various plants. Genome-wide analyses of miRNA have revealed that several miRNA families are highly conserved among plant genomes (conserved miRNAs), though individual species also possess highly specific and evolved (or evolving) miRNAs genes (novel miRNAs). It has been reported that conserved miRNA families have been expanded by duplication followed by subsequent reduction of redundant homologs whereas novel miRNAs, which are often expressed only in restricted species, might initially evolve neutrally but develop more specialized roles [27-32]. Sequence similarity-based approaches can be used to identify conserved miRNA candidates from newly-sequenced genomes. However, due to their species-specific nature, it is difficult to identify novel miRNAs by computational or hybridization-based methodologies. Recent advances in high-throughput sequencing technologies have facilitated the discovery of both conserved miRNAs and the less abundant novel and non-conserved miRNAs in plants [30,33-36]. Despite their importance in plant development, the genomic origin and evolution of miRNAs in polyploid genomes have not been well-described.

*Brassica rapa*, one of the two ancestral species of oilseed rape, is a member of the Brassicaceae family, which includes the model plant *Arabidopsis thaliana* and over 300 other genera. *B. rapa* shows great morphological plasticity, which has led to its domestication and selective breeding into a range of different crop types, such as Kimchi cabbage (Korean heading form), turnip, Mizuna, and rape mustard. This morphological diversity makes *B. rapa* an excellent species for the study of plant morphology evolution as well as the process of domestication and directed selection. An annotated draft genome sequence for *Brassica rapa* ssp. *pekinensis* cv. *Chiifu* was recently reported by the multinational *Brassica rapa* genome sequencing project consortium [9]. Comparative genomic analyses showed that the *B. rapa* genome had extensive collinearity with the genome of *A. thaliana*, evident in its triplicated and rearranged genome blocks. The extent of gene loss (fractionation) varied between the related genome segments of *B. rapa*, with one copy containing a greater proportion of genes expected to have been present in its ancestor (70%) than the remaining two (36% and 46%) [37]. A rapid evolutionary rate and the specific copy number amplifications of particular gene families are believed to contribute to the remarkable morphological plasticity of *Brassica* species. Therefore, the *B. rapa* genome sequence provides an important resource for studying the evolution of polyploidy genomes and the epigenetic regulation of duplicate genes.

To study epigenetic control in a triplicated crop genome, the genome-wide identification of *B. rapa* miRNAs is highly essential. Recent studies have identified a limited number of small RNAs in *B. rapa.* Most of these sequences were conserved miRNAs based on computational similarity searches with restricted sequence datasets, with very few novel miRNAs identified [38-40]. The main objective of this study was to identify putative miRNA candidates of *B. rapa* using a draft of its whole genome sequence. We identified miRNAs based on small RNA deep sequencing along with similarity searches, and then characterized the genomic organization and evolutionary origin of the miRNAs. In addition, we predicted the target genes of the candidate miRNAs and analyzed the putative function of miRNA targets. We constructed a web interface for the public to access the data generated in this study in order to provide a resource for studying the epigenetic control of gene expression in *Brassica* species.

## Results

### Preparation of small RNA dataset

In this study, we tried to identify miRNAs from the whole genome of *B. rapa* based on small RNA sequencing along with similarity searches using conserved miRNAs. To identify the miRNAs expressed in *B. rapa*, we performed high-throughput small RNA sequencing of five libraries constructed from seedlings, roots, petioles, leaves, and flowers using Illumina GA IIx sequencing technology. The number of sequence reads (hereafter "reads") obtained from each library was different presumably because of the quantitative differences in small RNA expression in each tissue type. From the raw sequence reads, the 5’- and 3’- adapter sequences were computationally removed and the remaining reads of 17–36 nt in length were collected. After collapsing redundancies, we obtained a total of 9,130,544 unique small RNA sequence reads from the five libraries (Table 1). The size distribution of the unique reads in each tissue is summarized in Additional file 1: Figure S1. Small RNAs of 24 nt in length were the most abundant class among the sequences, accounting for 41% (petiole) to 54% (seedling) of all the small RNAs. This result was consistent with previous reports for other plant species such as *A. thaliana*[30], *Medicago truncatula*[33], and *Oriza sativa*[41], where the 24 nt class dominated the small RNA transcriptome. The second most abundant small RNA in all five libraries was the 21 nt class, which covered approximately 7% to 9% of the unique reads and were the typical length of plant mature miRNAs.

**Table 1:** **Statistics of the small RNA sequences generated from the five tissue types of *B. rapa***

	**Seedling**	**Root**	**Petiole**	**Leaf**	**Flower**	**Total**
**Raw reads**	258,431	22,729	17,263,448	21,372,578	8,737,275	47,654,461
**Reads of 17 to 36 nt**	243,640	21,680	11,813,169	20,598,405	8,124,108	40,801,002
**Unique reads**	156,651	17,771	2,219,348	5,150,968	2,925,823	9,130,544^b^
**Genome match**^**a**^	51,147	5,184	509,173	1,575,267	1,106,694	2,752,756^b^
**Unassembled sequence match**^**a**^	60,937	6,638	1,049,984	1,686,965	919,182	3,139,339^b^
**NCBI NR database match**^**a**^	8,514	1,003	122,912	352,798	153,372	577,500^b^
**No match**^**a**^	36,053	4,946	537,279	1,535,938	746,575	2,660,949^b^

^a^ one mismatch is allowed, ^b^ non-redundant unique reads.

### Identification of *B. rapa* miRNA genes

**Figure 1:**
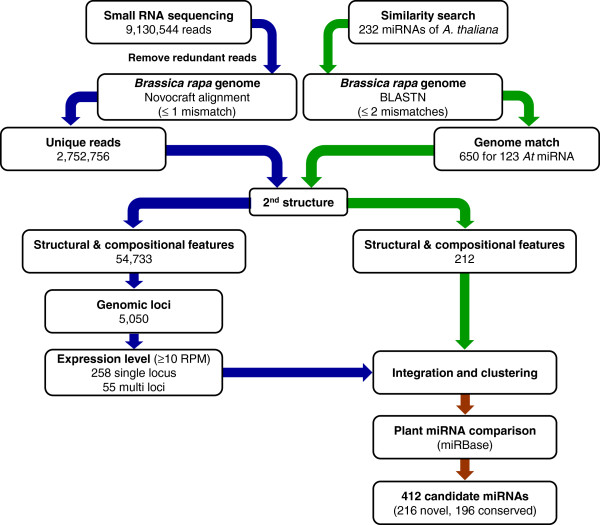
**Workflow to identify *B. rapa *miRNA genes.** Candidate miRNA genes were predicted based on a combination of small RNA deep sequencing (blue arrows) and similarity searches using known *A. thaliana* miRNA sequences (green arrows). Sequence reads generated from small RNA sequencing were aligned to the draft genome sequences of *B. rapa* followed by secondary structure modeling. The resulting reads were further analyzed for structural and compositional features, genomic position, and expression level validation. Similarity searches using known *A. thaliana* miRNAs followed by secondary structure modeling and structural and compositional feature analysis identified matches of conserved miRNA candidates in the *B. rapa* genome. Integration and clustering of both data sets resulted in the final miRNA candidate genes (brown arrows).

To predict *B. rapa* miRNAs from the sequencing data, we followed the procedure shown in Figure 1. In general, the small RNA libraries generated for sequencing have complex RNA compositions. In addition to miRNAs and siRNAs, the libraries contain large numbers of intermediate forms of small RNAs, rRNAs, tRNAs, and fragments derived from other coding and noncoding transcripts [42,43]. Therefore, to differentiate the small RNAs from the contaminants, we matched the unique reads to the reference genome sequences of *B. rapa* using a Novocraft aligner package with high stringency (one mismatch allowed). Approximately 30% of the unique reads were mapped to the reference genome sequences, representing 2,752,756 unique small RNA reads from 3,384,815 loci. In addition, 40% of the reads were mapped to unassembled genome sequences (34%) or sequences in the NCBI nucleotide database (6%), suggesting that they were transcribed from genomic regions that are not covered by the current reference genome sequences of *B. rapa*. The remaining 29% of the reads did not match any of the available sequences, presumably due to sequencing error or RNA modifications. This anticipation is based on the fact that approximately 97% of small RNA reads matched the available sequences under less stringent conditions (two mismatches allowed), (Additional file 2: Table S1). In addition to the small RNA sequencing, similarity searches also identified known *B. rapa* miRNAs that are evolutionary conserved within the miRNAs of *A. thaliana*. Because only mature miRNAs are usually conserved in plants [44], we used 232 mature miRNAs of *A. thaliana* downloaded from the miRBase [45] to find conserved miRNAs in *B. rapa* through sequence comparison. BLASTN searches against the *B. rapa* genome predicted 650 genomic loci that matched 123 *A. thaliana* miRNAs.

The miRNA prediction procedure using pre-processed reads was based on the structural features of the miRNA and precursor sequences, as well as the expression levels obtained from the sequencing data. Because miRNAs are derived from hairpin-like precursors originating from a single-stranded RNA transcript after sequential processing by Dicer or DCL proteins [46], miRNA precursors should have a characteristic fold-back structure. To identify candidate precursor from false positives, we used a sequence and structural feature filter designed based on *A. thaliana* miRNAs and their precursors. Putative precursors were regarded as positive miRNA candidates if they met a set of criteria based on GC content, the size of the hairpin-loop, the number of hairpin-loops, the size of the bulge-loop, the number of bulge-loops, and the negative minimal folding free energy (MFE) of the hairpin. We searched potential precursor sequences surrounding 3,384,815 loci of 2,752,756 unique reads obtained from small RNA sequencing, as well as the 650 loci matched to 123 *A. thaliana* miRNAs. A total of 54,733 loci from the small RNA sequencing data initially passed the structural feature filter. After removing sequences from redundant genomic positions, this number was collapsed to 5,050 loci. To better identify true miRNA candidates, expression levels were also analyzed. Considering the potential for sequence errors, sequences with fewer than 10 reads per million (RPM) in the total read counts from the five libraries were discarded. Afterward, sequences from 313 loci (55 multi-loci and 258 single-locus types) remained as miRNA candidates from the small RNA sequencing data. These sequences were integrated with the 212 conserved miRNA sequences identified based on the comparison with *A. thaliana* miRNA sequences that also passed the structural feature filter. All candidate miRNAs were clustered based on their sequences and genomic positions, and then cross-referenced with known plant miRNAs sequences in the miRBase. In total, we identified 412 miRNA candidates predicted to be in the *B. rapa* genome, of which 196 are conserved with miRNAs from other species while 216 represent novel miRNA sequences (Figure 1 and Additional file 3: Table S2).

### Prediction of miRNA target genes

**Figure 2:**
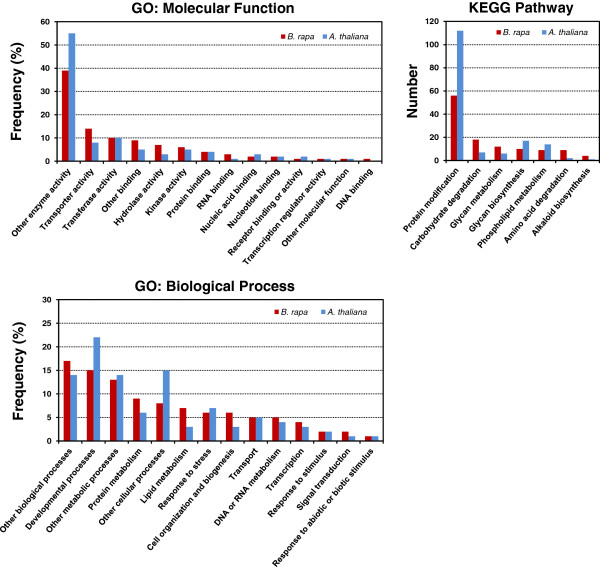
**Functional classification of miRNA target genes in *B. rapa *and *A. thaliana *based on gene ontology mapping using GO molecular function and GO biological process term databases, and pathway mapping using the KEGG pathway database.**

Identifying the genes targeted by miRNAs is crucial to understanding their biological functions. We searched the transcribed regions of the *B. rapa* genome to find sequences complementary to the miRNA candidates. A total of 8,199 genes (approximately 20% of protein coding genes) were predicted to be potential targets of the 412 miRNA candidates (Additional file 3: Table S2). This is consistent with previous reports from plant [47] and animal genomes [48]. Conserved miRNAs had nearly twice as many target genes as novel miRNAs (28.9 target genes/conserved miRNA versus 15.2 target genes/novel miRNA, respectively). To better understand the functional roles of the predicted miRNA targets, we performed functional annotation of the target genes based on gene ontology (GO) in molecular function and biological process, and metabolic pathway mapping using the KEGG pathway [49] (Figure 2). Molecular function was classified into 14 reference terms, with the most frequent terms being “other enzyme activity,” followed by “transporter,” “transferase activity,” “other binding,” “hydrolase activity,” and “kinase activity.” Biological process was also classified into 14 reference terms. The five most frequent terms were “other biological processes,” “developmental processes,” “other metabolic processes,” “protein metabolism,” and “other cellular processes.” In addition, the target enrichment test of the miRNAs using metabolic pathways demonstrated that “protein modification” was the most represented pathway followed by “carbohydrate degradation,” “glycan metabolism,” “biosynthesis,” and “phospholipid metabolism.” Interestingly, almost 12% and 7% of the putative target genes of miRNAs encoded transcription factors and kinases, respectively, suggesting that miRNAs may play important roles in regulating transcriptional and post-transcriptional networks and signaling processes. Although the functional classification of miRNA target genes in *B. rapa* showed a similar pattern of GO categorization as the miRNA targets in *A. thaliana*, several categories were under- or over-represented in *B. rapa*. For example, *B. rapa* miRNAs had a reduced number of target genes with “other enzyme activity” or involving the “developmental process,” “other cellular processes,” and the “protein modification” pathway. On the other hand, *B. rapa* miRNAs had approximately two times as many predicted target genes with “transporter activity,” “hydrolase activity,” “RNA binding,” and “lipid metabolism” compared to *A. thaliana* miRNAs. There were no functional differences between the target genes of conserved and novel miRNAs in *B. rapa* (data not shown).

### Expression profiles of *B. rapa* miRNAs

**Figure 3:**
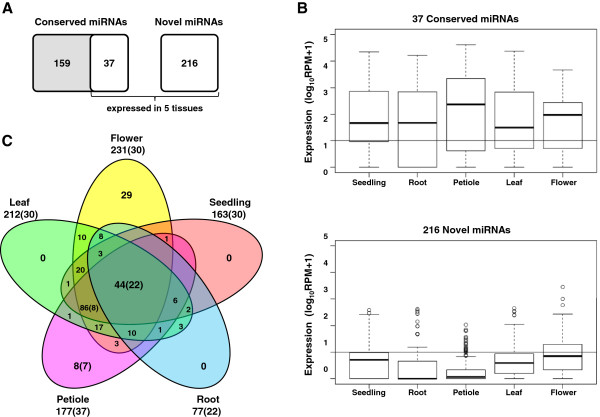
**Expression profiles of miRNA genes in *B. rapa ***. (**A**) Venn diagram summarizing conserved and novel miRNAs identified in *B. rapa*. (**B**) Boxplots of expression levels for 37 conserved miRNAs and 216 novel miRNAs in the five tissue types. Expression levels are presented as the log_10_ ratio of normalized RPM. (**C**) Venn diagram showing the number of miRNAs expressed in specific tissues. The numbers in parentheses refer to the conserved miRNAs.

Quantitative analysis of miRNA expression revealed that only 37 members of the conserved miRNAs were expressed in the five types of *B. rapa* tissue we examined (Figure 3A). However, their expression levels were significantly higher than those of the novel miRNAs. While the expression levels among all the miRNAs ranged from 10 to 73,004 RPM, the mean RPM value of conserved miRNAs (5,031 RPM) was approximately 60 times higher than that of novel miRNAs (85 RPM) (Figure 3B). This finding is consistent with previous studies reporting that evolutionarily conserved miRNAs were often among the most abundant miRNAs, and that some non-conserved miRNAs may be in low abundance because they recently evolved in specific species [32]. The expression profiles from the different tissue types also showed that 44 miRNAs were expressed in all five types of tissue, suggesting that they are ‘housekeeping’ miRNAs important for regulating basic cellular functions in all tissues.

Currently, the miRBase lists 23 *B. rapa* miRNAs that can be grouped into 10 miRNA families. Our prediction methods successfully identified nine of the 10 families (it did not identify miR1885). Surveying the expression levels of these families shows that most of the known miRNA families are expressed in all five tissue types (Additional file 3: Table S2). Moreover, a total of 179 novel miRNAs appeared in multiple libraries, though the flower and petiole libraries included 29 and eight tissue-specific miRNAs, respectively (Figure 3C and Additional file 4: Table S3). Of particular interest, all the flower-specific miRNAs were novel and had target genes that include transcription factors and kinases related to flowering, suggesting that they may play roles in the development of floral organs. In addition, most of the petiole-specific miRNAs were members of the conserved miRNA families miR164 and miR167, although there was one novel miRNA (bra-miR6104). Both the miR164 and miR167 families were identified as stem-specific miRNAs from *A. thaliana* and tobacco [50,51].

### Synteny comparison of miRNA genes between *B. rapa* and *A. thaliana*

**Figure 4:**
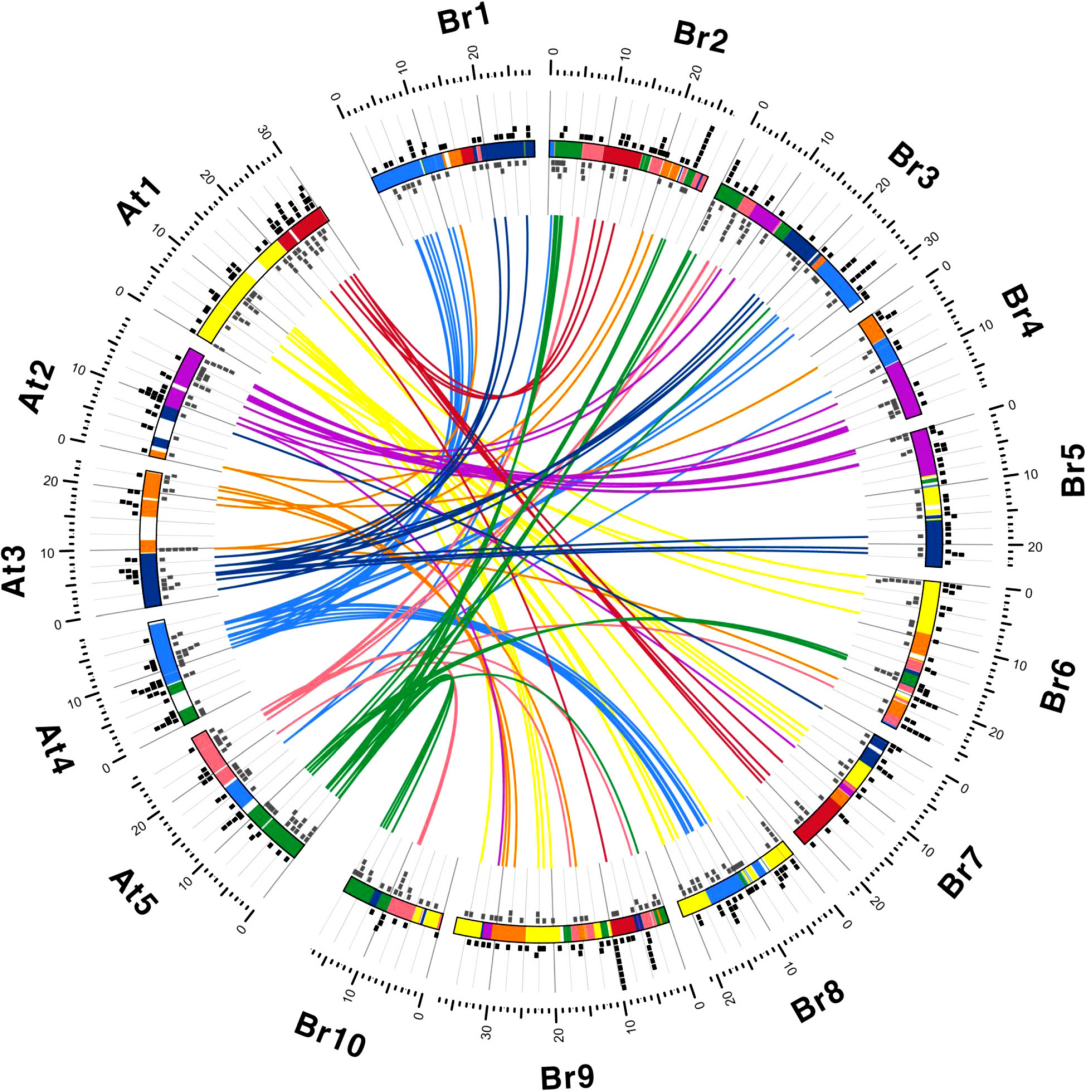
**Circos diagram of miRNA gene pairs between *B. rapa *and *A. thaliana*.** Conserved miRNAs genes in *B. rapa* are plotted against their syntenic counterparts in *A. thaliana*. The individual chromosomes of *B. rapa* (Br) and *A. thaliana* (At) have 24 ancestral karyotype genome building blocks demonstrating the shared ancestral origin of their genomes. The black and gray dots represent novel and conserved miRNA genes, respectively, of each genome within a 500 kb interval. The syntenic counterparts of conserved miRNAs between the genomes are interconnected by colored lines.

Comparing the genomic positions of miRNA genes in *B. rapa* and *A. thaliana* provides insight into the origin and evolution of *B. rapa* miRNA genes. Considering its shared ancestry with *A. thaliana* and its recent WGT event, the *B. rapa* genome was predicted to contain approximately 300 conserved miRNA genes. However, we could identify only 196 conserved miRNA genes in the *B. rapa* genome. To investigate this discrepancy, we compared syntenic blocks between the two genomes. A Circos plot between orthologous miRNA gene pairs revealed that 140 of the 196 *B. rapa* miRNA genes had their syntenic counterparts in the same ancestral karyotype genome building blocks of the *A. thaliana* genome, indicating that the conserved miRNA genes share a common ancestry (Figure 4). In general, syntenic relationships were found among the conserved miRNA families, with the exception of miR482, miR528, miR529, and miR1432, which are absent in *B. rapa*. Interestingly, most of the conserved miRNA genes in *B. rapa* were present in only one or two copies relative to their *A. thaliana* miRNA counterparts. This suggests that the deletion of one or more redundant paralogs has occurred throughout the *B. rapa* genome since its WGT. In addition to sharing conserved miRNA genes, it was evident that more than 50% of the miRNA genes of *B. rapa* and *A. thaliana* were species-specific in each genome. The genomic position of miRNA genes showed that they mainly resided in the intergenic regions (86% in *B. rapa* and 75% in *A. thaliana*), with several clusters on chromosomes A2, A3, A6, and A9 of *B. rapa* and chromosomes 1 and 4 of *A. thaliana*.

### Database access

**Figure 5:**
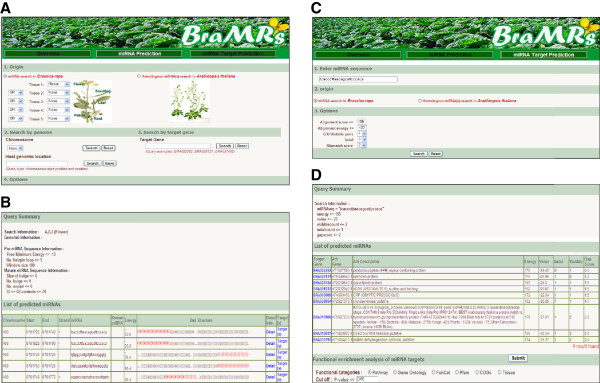
**Screenshots of sample output pictures from the BraMRs database.** (**A**) Snapshot of the “miRNA Prediction” unit providing search options for different tissues, chromosomes, and target genes. (**B**) An example of search results from the “miRNA Prediction” function presenting a list of predicted miRNAs with relevant sequence information and links to the target gene and expression characteristics. (**C**) Snapshot of the “miRNA Target Prediction” unit for searching for the putative target genes of the miRNA. (**D**) An example of a search result from the “miRNA Target Prediction” function presenting a list of predicted miRNA target genes with information on the binding site, alignment score, number/type of mismatches, and links to putative function and EST matches.

We developed the BraMRs [1] web interface for the public to access the *B. rapa* miRNAs dataset. This database integrates all the data generated in this study, including *B. rapa* miRNA identification, target gene prediction, and functional annotation for the targets. Figure 5 provides examples of database searches and search outputs from a web implementation of BraMRs. BraMRs consists of two major search units, “miRNA Prediction” and “miRNA Target Prediction,” which are tightly inter-connected. The main search page of “miRNA Prediction” allows the user to search two classes of miRNAs, those with supporting expression data (expressed type) and those predicted based on sequence conservation (conserved type). The user can identify miRNAs based on their chromosomal or genomic position, or by their predicted target gene. Various filtering options for miRNA predictions can be customized by the user (Figure 5A). The query result displays a list of predicted miRNAs with links to miRNA and target gene sequences (Figure 5B). The search results also include both the genomic position of the miRNA along with the secondary structure of candidate pre-miRNAs and the miRNA expression levels in the five tissue types. Each predicted miRNA is also linked to a page of potential miRNA targets. Alternatively, the user can directly identify putative miRNA targets by providing a miRNA sequence in the “miRNA Target Prediction” window. The stringency of this search can be customized by adjusting various parameters affecting the alignment (Figure 5C). The output page lists putative target genes, miRNA binding sites, alignment scores, and the number/type of mismatches (Figure 5D). Importantly, BraMRs facilitates the functional enrichment analysis of predicted miRNA targets, allowing the user to develop hypotheses regarding miRNA function. Links to Pfam domain information and expressed sequence tag (EST) records are also provided.

## Discussion

The progress being made in genome sequencing has allowed for the identification of the miRNAs of various plant genomes ranging from mosses and ferns to flowering plants. One popular strategy used to predict potential miRNAs from plant genomes is to run similarity searches based on previously identified miRNAs. As a result, a large number of miRNAs and their target genes are available in public databases such as the miRBase [45] and the PMRD [52]. However computational methods often identify only evolutionary conserved miRNAs and sometimes candidate miRNAs are misannotated due to incomplete sequences and the lack of expression data. Several previous studies have identified *B. rapa* miRNAs using computational approaches. For example, Dhandapani et al. (2011) reported 186 miRNAs while Wang et al. identified 168 miRNAs, all of which were evolutionarily conserved [39,40]. In contrast to these computational approaches, high-throughput sequencing of small RNAs using next-generation sequencing technology provides more reliable predictions of miRNAs based on empirically-derived expression data, enabling researchers to discover many recently-evolved novel miRNAs. In the current study, we successfully identified conserved miRNAs as well as a large number of novel miRNAs from the *B. rapa* genome using a combination of small RNA deep sequencing and similarity-based miRNA predictions. A total of 412 miRNAs were identified, of which 216 were novel miRNAs. This is the first report to identify novel miRNAs from *Brassica* crops using high throughput methods. Considering that the draft genome sequence of *B. rapa* covers only 50% of the span of the genome representing gene space [9] and that approximately 40% of small RNA reads can match to unassembled genome sequences, additional novel miRNAs can be identified from the genomic regions that has not yet been characterized.

Recent studies have demonstrated that evolutionary conserved miRNAs exist as gene families whereas recently evolved or evolving miRNAs are frequently found to have a single locus in the genome [30,53]. Based on the identification of conserved miRNAs across multiple species, it has been suggested that the gene regulation mediated by conserved miRNAs is ancient, systemic, and shared in many plant genomes. Recently evolved novel miRNAs, on the other hand, are present in relatively low abundance and are expressed in specialized tissues [36]. These observations are consistent with our study, which found that 88% of conserved miRNAs existed as gene families whereas 76% of novel miRNAs were found to have a single locus in the *B. rapa* genome (Additional file 3: Table S2). Furthermore, our quantitative expression analysis indicated that almost 14% of the novel miRNAs (30 of 216) were expressed in a single tissue compared to only 4% of the conserved miRNAs (8 of 196). Approximately 10% of both the conserved (22 of 196) and the novel miRNAs (22 of 216) were expressed in all of the tissue types investigated. We compared the genomic position of the miRNA genes of *B. rapa* and *A. thaliana* and found that approximately 50% of the miRNA genes are shared between the genomes and the remaining 50% are specific to each species. These species-specific miRNAs are dispersed throughout the genomes and enriched within the intergenic region. Similar findings have also been reported when comparing the genomes of related species of legumes and grasses. For example, 29% of rice miRNAs (189 of 661) are conserved in maize, while only 19% of *M. truncatula* miRNAs (125 of 674) have homologs in soybean. These findings suggest that novel miRNAs have independently emerged in plant genomes after speciation. Several different mechanisms have been proposed for this miRNA evolution, including the duplication of protein coding genes, the duplication of pre-existing miRNA genes, and the modification of transposons [54]. The *B. rapa* genome contains a large number of repetitive sequences including MITEs and inverted repeats. The modification of these sequences may give rise to hairpin structures which can become new miRNA genes. Considering the fact that most plant genomes have experienced transposon invasion, the abundance of novel miRNA genes in each plant genome strongly suggests that miRNA gene repertories rapidly change during genome evolution, lending credence to a birth and death model of miRNA gene evolution.

The identification of a large number of novel miRNAs in *B. rapa* provides an opportunity to study the unique epigenetic control of target genes. The expression patterns of miRNAs have been reported in multiple plant species and are regulated by development, tissue type, and stress treatment [12,19-22]. It is noteworthy that the 29 novel miRNAs we identified showed flower-specific expression. Consistent with previous reports, most of the genes targeted by these novel miRNAs included transcription factors, protein kinases, and genes related to signaling (Additional file 4: Table S3). For example, miR6004*, miR6093, and miR6020 could target transcription factors related to flowering including *Agamous-like* (*AGL*), *vernalization factor 3*, and *MADS affecting flowering 3*. The up-regulation of these novel miRNA genes in flowers could suppress the corresponding target genes during flower development. Therefore, the comprehensive functional analysis of these miRNAs and their target genes can provide novel insight into miRNA-mediated epigenetic control during the development of flowers and reproductive tissues in *B. rapa*.

## Conclusions

This work has contributed to an increased insight into the *B. rapa* genome. Deep sequencing of small RNAs has proven to be an effective approach that allows for the genome-wide discovery of novel miRNAs in *B. rapa*. The finding of numerous novel miRNAs, many with few target genes and low expression levels, suggests the ongoing birth and death of miRNA genes. The systematic characterization of small RNAs from additional tissue types and developmental conditions will further enrich this miRNA collection and provide a valuable public resource for studying the epigenetic control of the *Brassica* genomes. Furthermore, the functional study of novel miRNAs will increase our understanding of the epigenetic regulation of polyploid genomes. In this regard, BraMRs, the *B. rapa* miRNA database developed here, will provide fundamental information for the epigenetic study of *B. rapa* and other closely related species.

## Methods

### Reference sequences and plant material

To identify miRNAs and their targets, we used the draft genome sequence v1 of *B. rapa* ssp. *pekinensis* cv. *Chiifu* along with 41,174 annotated gene models [9]. For a reference set of miRNAs, we downloaded known mature plant miRNAs and their precursor sequences from the miRBase release 17 [55] as of August 2011. The seeds of *B. rapa* ssp. *pekinensis* cv. *Chiifu* were surface-sterilized in 12% sodium hypochlorite and germinated on 0.5X Murashige and Skoog (MS) agar plates (0.7%) in a growth chamber at 22°C and a 16h light/8h dark cycle with 60% humidity. *B. rapa* seedlings were either harvested seven days after germination or moved into soil and grown under the same conditions described above. Vegetative tissues from the root, petiole, and leaf were harvested from one-month-old plants. To collect floral tissues, one-month-old plants were vernalized for four weeks at 4°C and subsequently grown in a greenhouse. Flowers were harvested prior to anthesis.

### Library construction, small RNA sequencing, and preprocessing of data

Total RNA was isolated from the plant tissues using a GeneAll HybridR^+^ kit (GeneAll, Seoul, Korea) and low molecular weight RNA was purified using a DGE-Small RNA Sample Preparation Kit (Illumina, San Diego, USA) according to the manufacturer’s instruction. Purified small RNAs were ligated to adapters and underwent reverse transcription, PCR amplification, and single-read cluster generation on an Illumina flow cell using cBot. The library clusters were then sequenced on an Illumina GA IIx sequencer using the TruSeq SBS kit v4-GA to generate 36 base pair (bp) single-end sequences. Before data analysis, the small RNA reads were pre-processed to remove low quality reads and to trim the adaptor sequences. Reads in which no adaptor sequences were detected were also discarded. The resulting 17–36 nt reads were collected using a Python script. To identify the miRNAs expressed in *B. rapa*, reads were aligned to the *B. rapa* reference genome sequences using the Novocraft aligner package version 2.07 [56] at a setting which allowed a single mismatch within each sequence. In order to identify conserved miRNA candidates in *B. rapa*, mature miRNAs of *A. thaliana* were mapped to the *B. rapa* genome sequences using a BLASTN search [57-60], allowing a maximum of two mismatches. Each genomic locus with short sequences was obtained by comparing the data from the small RNA sequencing and the similarity search. For quantifying the gene expression, the number of reads per miRNA candidate was normalized to RPMs against the total number of reads for each library, with the cutoff for read count set to 10 [55,61,62].

### Identification of miRNAs

The miRNA precursors were identified using the Vienna RNA package v.1.5.8 [63] by shifting a window across the genomic sequences surrounding each candidate miRNA and then folding a sequence within a window ranged from 70 to 300 nt (70, 100, 150, 200, 250, and 300 nt) while considering the length and structural features of pre-miRNAs in *A. thaliana*. The calculated structural features for the pre-miRNA of *A. thaliana* were defined as follows: secondary structure with one or two stem-loop hairpins, GC content ranging from 22% to 73%, stem-loop size ranging from 7 to 75 nt, bulge-loop size less than 7 nt, and MFE less than −15 kcal/mol with an average of −47.2 kcal/mol. Additionally, all of the *B. rapa* miRNA should have at least 76% sequence complementarity to their *A. thaliana* counterparts. The genomic positions of the miRNAs were compared and cases with more than 5 bp overlap were considered to be the same genomic loci. The sequences that met each of these criteria were considered to be candidate miRNA precursors.

### Prediction of miRNA targets and functional enrichment analysis

We searched for the target genes of miRNAs using the miRanda application (Sep. 2008 release) [64]. In order to reduce the rate of false positive target predictions, we used three stringent criteria: the miRNA complementarity score, the pairing and energy scores, and the miRNA:mRNA duplex pairing composition. Each complementary miRNA site was scored, with perfect matches given a score of 0 and points added for each G:U wobble (0.5 points) and non-G:U mismatch (1 point). Only the candidate miRNAs with at least one predicted target with a score ≤ 3.0 were considered. The cutoff of the miRanda pairing score of the miRNA:mRNA duplex was set to 156 and the cutoff of the MFE was set to −20 kcal/mol. The pairing composition of the miRNA:mRNA duplex was based on the following rules: no mismatches at positions 10 and 11, no more than one mismatch at positions 2 to 12, and no more than two consecutive mismatches downstream of position 13 [65]. To understand the putative roles of potential miRNA target genes, functional enrichment analysis was performed with GO mapping using the GO molecular function and GO biological process term databases, in addition to metabolic pathway mapping using the KEGG pathway database [49]. A hypergeometric distribution statistic was used to ensure that target genes were not hitting their corresponding molecular function and biological process categories by random chance. For multiple hypotheses tests, we applied the Benjamin and Hochberg false discovery rate (FDR) method to reduce false negatives to a Q-value < 0.2.

### Synteny comparison between *B. rapa* and *A. thaliana*

Syntenic regions between the *B. rapa* and *A. thaliana* genomes were identified by a proteome comparison based on BLASTP analysis, as described in our previous report [8]. The entire proteomes of the two genomes were compared, with only the top reciprocal BLASTP matches per chromosome pair selected (minimum of 50% alignment coverage at a cutoff of < E^-20^). Chromosome scale synteny blocks were inferred by the visual inspection of OSfinder v1.4 [66]. The assignment of 24 genome building blocks of the ancestral karyotype of both genomes was carried out according to previous comparative genome mapping studies [67,68]. Homologous miRNA gene pairs between the genomes were illustrated by Circos [69].

### Database construction

The *B. rapa* miRNA database, BraMRs, was constructed using a Mysql V.5.1.32 database engine on a server managed by the Red Hat 4.3.2 operating system. The scripting language PHP V5.2.9 was used to connect the database and web browser, and Apache V2.0 was used to display outputs of the queries on the web. The database is available at the following link: http://bramrs.rna.kr[1].

## Abbreviations

bp: Base pair; EST: Expressed sequence tag; FDR: False discovery rate; GO: Gene ontology; MFE: Minimal folding free energy; mRNA: Messenger RNA; miRNA: MicroRNA; MS media: Murashige and Skoog media; nt: Nucleotide; PCR: Polymerase chain reaction; pre-miRNA: Precursor microRNA; pri-miRNA: Primary transcript microRNA; RISC: RNA-induced silencing complex; RPM: Reads per million; siRNA: Short-interfering RNA; WGT: Whole genome triplication.

## Competing interests

The authors declare that they have no competing interests.

## Authors’ contributions

JHM conceived the project, designed research, and wrote the manuscript. BJK analyzed data, developed the BraMRs database, and participated in manuscript preparation. HJY conceived the project, designed research, performed small RNA sequencing, and participated in manuscript preparation. SGP, JYS, MO, and NSK contributed to data analysis and database development. All authors read and approved the final manuscript.

## Supplementary Material

Additional file 1 **Figure S1.** Size distribution of unique small RNA sequence reads obtained from the five tissue types (seedlings, roots, petioles, leaves, and flowers) of *B. rapa* using Illumina GA IIx. Click here for file

Additional file 2 **Table S1.** Statistics of the small RNA sequence matches on the *B. rapa* genome under two-mismatch condition. Click here for file

Additional file 3 **Table S2.** Summary of miRNAs identified in this study. The miRNA sequences and their genomic positions with target genes and expression levels in the tissues are presented. Click here for file

Additional file 4 **Table S3.** Flower- and petiole-specific miRNAs and their target genes. Click here for file
