# Supplementary figures and images for: Identification and profiling of novel microRNAs in the Brassica rapa genome based on small RNA deep sequencing
Source: BMC Plant Biol. 2012 Nov 19;12:218. doi: 10.1186/1471-2229-12-218 (PMC3554443; doi:10.1186/1471-2229-12-218)

## Slide 1
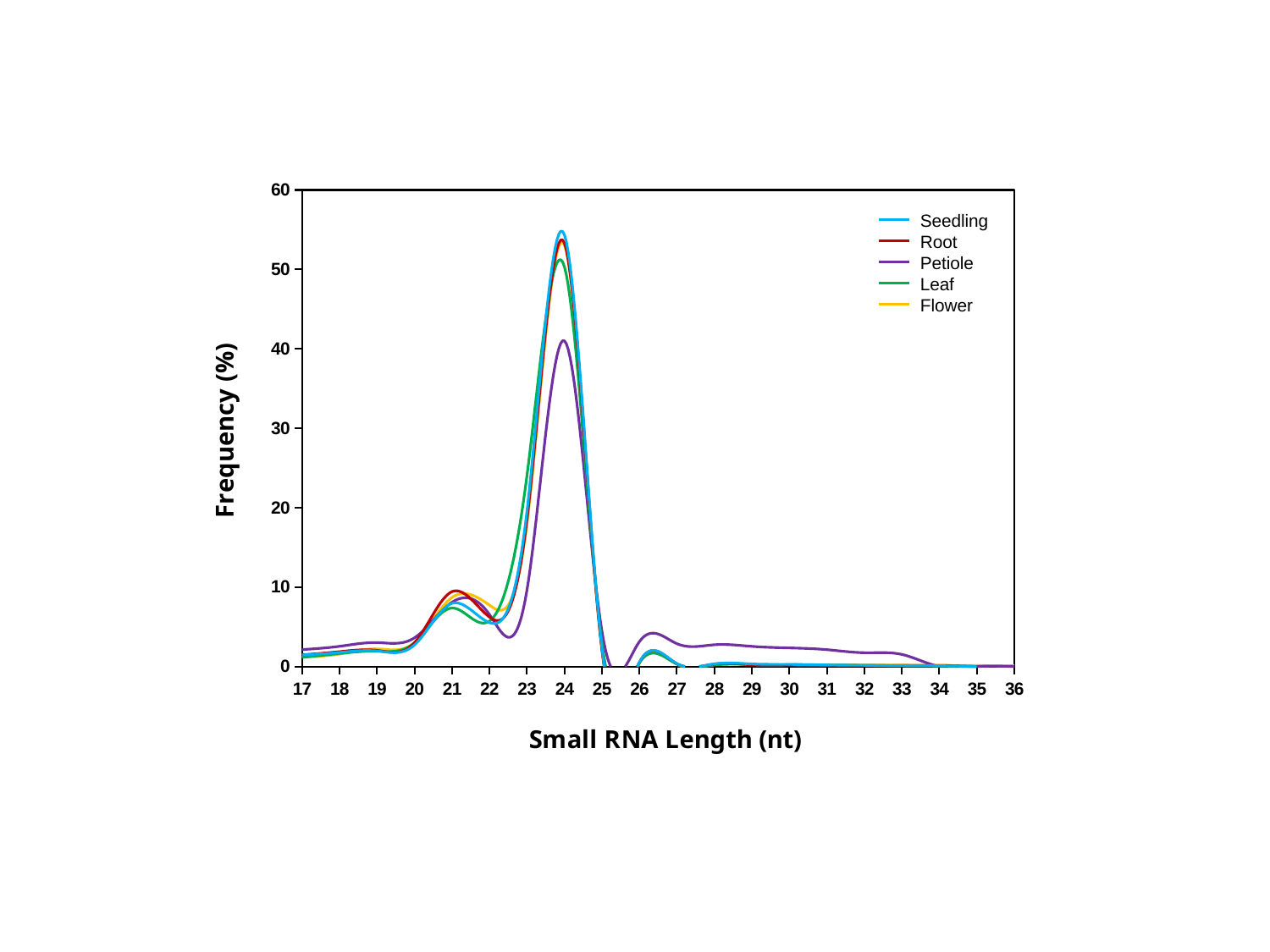

### Chart
| Category | seedling | root | leaf | stem | flower |
|---|---|---|---|---|---|Seedling
Root
Petiole
Leaf
Flower

Supplement: Additional file 1 — Figure S1. Size distribution of unique small RNA sequence reads obtained from the five tissue types (seedlings, roots, petioles, leaves, and flowers) of B. rapa using Illumina GA IIx. [file 1471-2229-12-218-S1.pptx]
