# Supplementary material for: Identification and profiling of novel microRNAs in the Brassica rapa genome based on small RNA deep sequencing
Source: BMC Plant Biol. 2012 Nov 19;12:218. doi: 10.1186/1471-2229-12-218 (PMC3554443; doi:10.1186/1471-2229-12-218)
Supplement: Additional file 2 — Table S1. Statistics of the small RNA sequence matches on the B. rapa genome under two-mismatch condition. [file 1471-2229-12-218-S2.docx]

**Table S1.** Statistics of small RNA sequence matches on the *B. rapa* genome under two-mismatch condition.

|  | **Seedling** | **Root** | **Petiole** | **Leaf** | **Flower** | **Total^a^** |
| --- | --- | --- | --- | --- | --- | --- |
| **Unique reads** | 156,651 | 17,771 | 2,219,348 | 5,150,968 | 2,925,823 | 9,130,544 |
| **Genome match** | 80,535 | 8,462 | 871,990 | 2,796,123 | 1,706,259 | 4,574,758 |
| **Unassembled sequence match** | 64,434 | 7,030 | 1,108,240 | 1,846,877 | 984,166 | 3,604,474 |
| **NCBI NR database match** | 9,681 | 1,227 | 129,928 | 412,493 | 177,907 | 667,162 |
| **No match** | 2,001 | 1,052 | 109,190 | 95,475 | 57,491 | 284,150 |

^a^ non-redundant unique reads.
